# Long non-coding RNA Linc00205 promotes hepatoblastoma progression through regulating microRNA-154-3p/Rho-associated coiled-coil Kinase 1 axis via mitogen-activated protein kinase signaling

**DOI:** 10.18632/aging.203902

**Published:** 2022-02-17

**Authors:** Guoqing Liu, Qiang Zhu, Hao Wang, Jianfeng Zhou, Bin Jiang

**Affiliations:** 1 Department of General Surgery, Children’s Hospital of Nanjing Medical University, Nanjing 210008, China

**Keywords:** hepatoblastoma, miR-154-3p, ROCK1, MAPK signalling pathway

## Abstract

Hepatoblastoma (HB) is the most common pediatric liver tumor. The significant tumor heterogeneity of HB leads to varied prognoses among children with the disease. Recent studies have suggested that long non-coding RNAs (lncRNAs) can serve as novel therapies for HB treatment. Thus, in this study, we aimed to reveal the function and mechanism of the lncRNA Linc00205 in HB. Our results exhibited that, in both HB tissues and cell lines, levels of Linc00205 were significantly increased. In addition, knockdown of Linc00205 led to suppression of HB development. Moreover, we identified that Linc00205 was able to directly bind to miR-154-3p, thus isolating miR-154-3p from its target Rho-associated coiled-coil Kinase 1 (ROCK1). Further cellular behavioral experiments elucidated that the miR-154-3p inhibitor and ROCK1 overexpression were able to reverse the effect of downregulated Linc00205 on proliferation, migration, invasion, and apoptosis of HB cells by rescue assays via mitogen-activated protein kinase (MAPK) signaling. Our results demonstrated that Linc00205 enhanced HB progression by regulating ROCK1 expression via sponging miR-154-3p through MAPK signaling, which suggests a novel potential therapeutic target for HB.

## INTRODUCTION

Hepatoblastoma (HB) is the most common primary malignant tumor of the liver among children, as it accounts for over 1% of pediatric malignancies. In recent years, there has been an increase in the incidence of HB, which is thought to be related to a higher number of premature births. Furthermore, infants with a birth weight lower than 1500g are predisposed to developing HB [1]. HB presents as a solid focal tumor that reproduces the liver in an embryologic development state [2]. The primary curative treatment for HB is surgical excision, and chemotherapy is essential in order to shrink an unresectable tumor to becoming resectable, and decreasing surgery-related morbidity [3]. Children that are within the low-risk category can have an 80% 5-year survival rate after treatment. However, for children that are high risk or after a relapse, the survival rate decreases to 30-40% [4]. Therefore, there is a significant need to achieve a more detailed understanding of HB in order to develop more effective therapies to improve patient outcomes.

Long non-coding RNAs (lncRNAs) are RNAs that are longer than 200 nucleotides. As they are non-coding RNAs, lncRNAs do not possess an open reading frame that encodes protein [5]. LncRNAs are known to regulate a variety of biological processes, including cell proliferation and differentiation, through scaffolding protein-protein/DNA interaction, binding to protein or microRNAs (miRNAs), or enhancers of genes. As lncRNAs can have an effect on cell cycle, dysregulation of lncRNAs can be found across different types of cancers. Studies have suggested that lncRNAs can play a role in cancer initiation and progression [6]. Recently, long intergenic non-protein coding RNA 205 (Linc00205) have been found to promote malignancy in lung cancer by recruiting the RNA-binding protein FUS to stabilize CSDE1 mRNA, which enhances lung cancer cell proliferation, apoptosis, and migration [7].

MiRNAs are another class of non-coding RNAs, but are only 18-25 nucleotides in length. MiRNAs are known to regulate gene expression by binding target messenger RNAs (mRNAs) at the 3’UTR in order to initiate degradation or repression of translation [8]. On the other hand, miR-4510 was considered to be a tumor suppressor as it suppressed the Wnt/β-catenin pathway by targeting the glypican-3 (*GPC3*) oncogene, thereby decreasing HB cell line and HuH6 cell viability [9]. miR-154-3p is tightly related to numerous physiological and pathological processes, including diabetes [10], cardiac complications [11] and cancers [12]. However, the function of miR-154-3p in HB remains unclear.

The relationship between lncRNAs and miRNAs has been explored across many studies. The lncRNA-TUG1 was found to be upregulated in HB tissues and cell lines, and interact with miR-34a-5p as a sponge in order to inhibit mRNAs from binding to its targets. The target mRNA of miR-34a-5p is VEGFA, which is a crucial protein that is involved in pathological angiogenesis. Thus, TUG1 promotes tumor growth and angiogenesis in HB by preventing miR-34a-5p from regulating VEGFA, which can cause TUG1 to be a possible therapeutic target for HB [13]. Linc00205 has been recognized as a molecular sponge for miR-122-5p in HCC cell line to promote cell growth [14]. However, the concrete role of Linc00205 in HB remains to be investigated for deep understanding.

Rho-associated coiled-coil kinases (ROCKs) are serine/threonine kinases that are downstream effectors of the small GTPases RhoA, RhoB, and RhoC. There are two ROCKs, including ROCK1 and ROCK2, which are similar to each other. Depending on their cellular location, they can function differently by regulating cell proliferation, apoptosis, cell adhesion, motility, and contraction [15]. Many studies have focused on the role of ROCKs in cancer. Several somatic mutations in ROCK1 are present in human cancers, and all these mutations have enhanced kinase activity of ROCK1, as well as increased motility of tumor cells [16]. MiRNAs are able to regulate ROCK1 expression, and many miRNAs expression changes have been identified in cancer tissues. The miR-124 and miR-145 were both found to target ROCK1 in glioma cells, and inhibit glioma migration [17, 18]. Furthermore, in thyroid cancer cells, ROCK1 expression was found to be negatively correlated to miR-154-3p [19].

Herein, the function and related mechanisms of lncRNA Linc00205 in HB were explored in-depth, and the results may be valuable for HB therapy.

## RESULTS

### Linc00205 expression is significantly increased in HB

**Figure 1:**
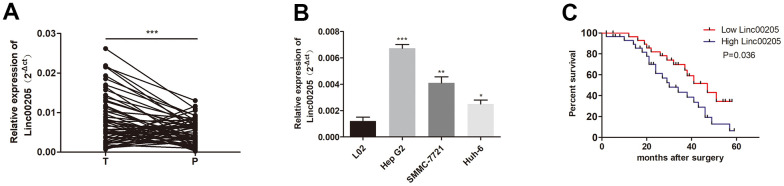
**Linc00205 expression in HB increased.** (**A**) qRT-PCR assay for Linc00205 expression among HB (n = 60) and para-cancerous tissues (n = 60). (**B**) qRT-PCR assay for Linc00205 expression in HB cell lines. (**C**) Kaplan-Meier curve for survival after surgery with low or high Linc00205 expression. *p < 0.05, **p < 0.01, ***p < 0.001.

We performed qRT-PCR assays in order to determine the expression of Linc00205 in HB tissues and para-cancerous normal tissues collected from 60 patients. The expression of Linc00205 in HB tissues was found to be remarkably higher compared to adjacent non-tumor tissues (Figure 1A; ***p<0.001). In addition, increased Linc00205 expression was observed in the three HB cell lines (Figure 1B; ***p<0.001, **p<0.01, *p<0.05). Furthermore, Kaplan-Meier survival analyses indicate that among the 60 patients, patients with low Linc00205 expression in their HB tissues were found to have a much higher five-year survival rate after surgery (Figure 1C, p=0.036). The results indicated that dysregulation of Linc00205 expression may play an important role in HB malignancy.

### Regulating Linc00205 expression can have an effect cell viability, proliferation, apoptosis, migration, invasion, and tumor formation

**Figure 2:**
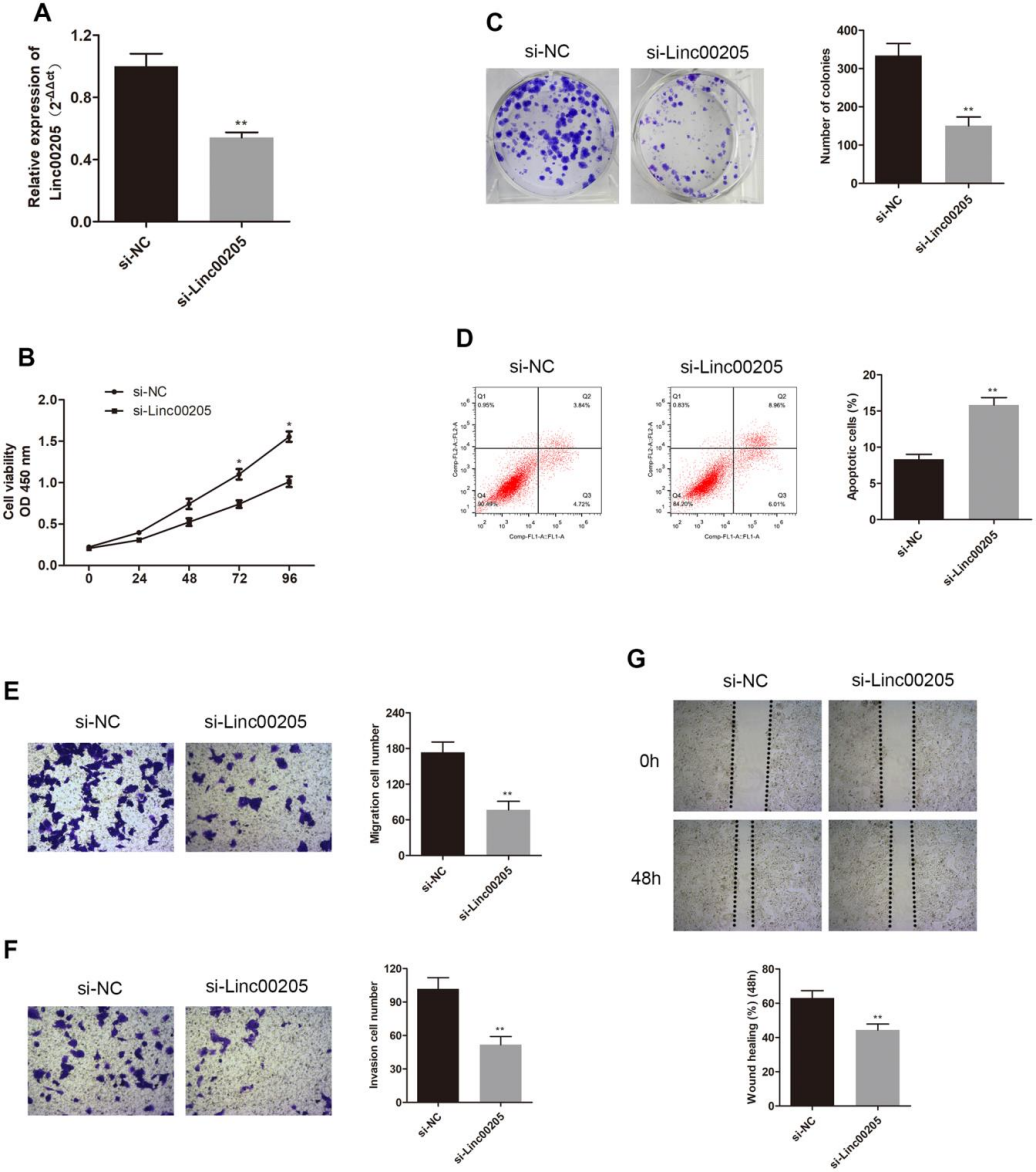
**Knockdown of Linc00205 altered cell viability, colony formation, apoptosis, migration and invasion ability.** (**A**) qRT-PCR assay for Linc00205 expression in HepG2 cells that were transfected with either si-Linc00205 or control si-NC. (**B**) Cell viability assay conducted in HepG2 cells after transfection with either si-Linc00205 or control si-NC. (**C**) Colony formation ability of HepG2 cells that were transfected with si-Linc00205 or control si-NC. Quantitative analysis of colonies formation (right panel). (**D**) Apoptosis of HepG2 cells transfected with si-Linc00205 or control si-NC, and quantitative analysis of the apoptosis cells (right panel). (**E**, **F**) The migration and invasion ability of HepG2 cells that were transfected with either si-Linc00205 or control si-NC, as determined using the transwell migration assay (**E**) and transwell invasion assay (**F**) (left panel). Quantitative analysis of the migratory cells or invasive cells in transwell assays was also conducted (right panel). (**G**) Migration ability of HepG2 cells transfected with si-Linc00205 or control si-NC at indicated time points was evaluated by wound healing assay (upper panel). Quantitative analysis of the gap of wound healing assay (lower panel) was performed at 48 hours. *p < 0.05, **p < 0.01.

**Figure 3:**
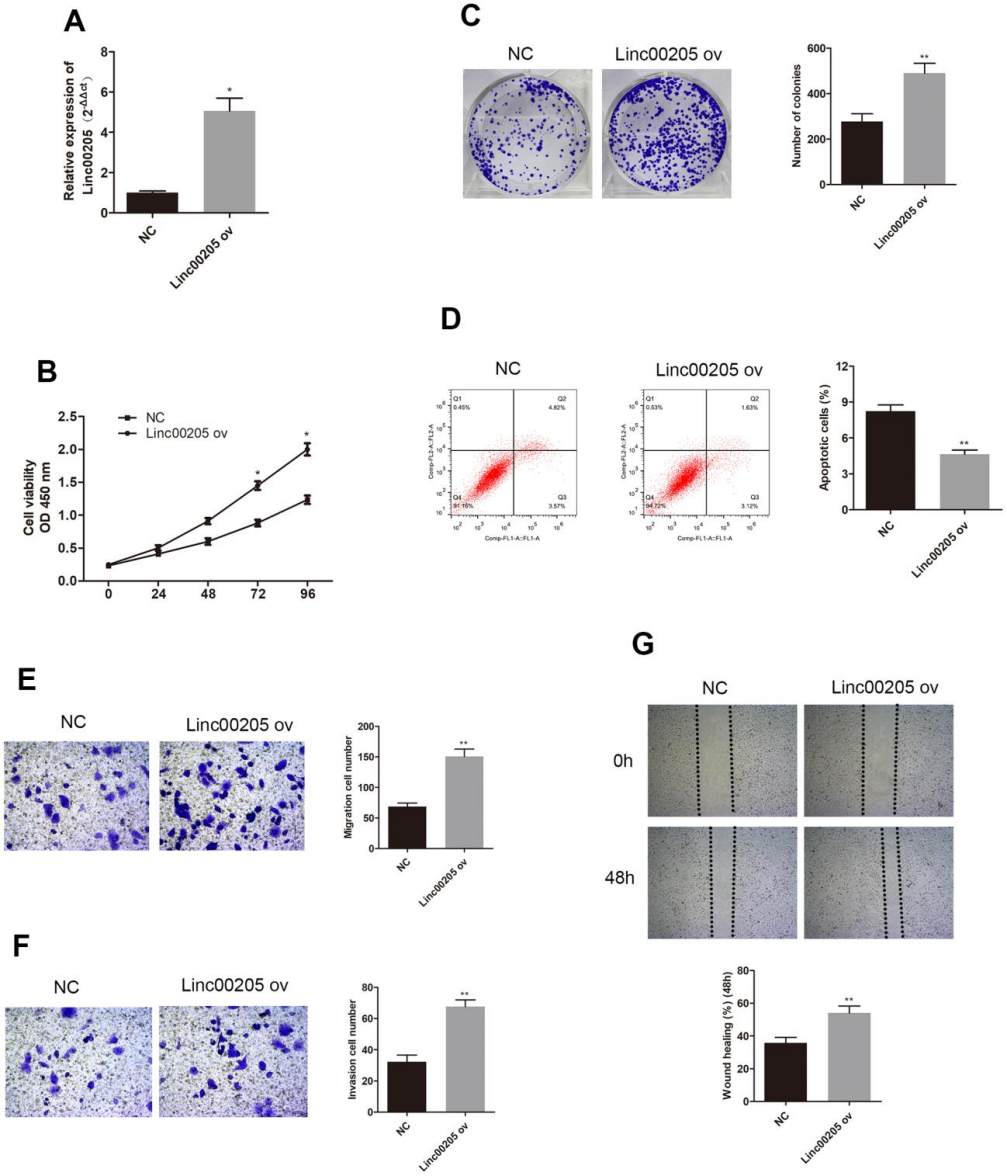
**Overexpression of Linc00205 altered cell viability, apoptosis, colony formation ability, apoptosis, migration and invasion ability.** (**A**) qRT-PCR assay for Linc00205 expression in Huh-6 cells transfected with Linc00205 ov expression (ov) or NC. (**B**) Cell viability assay was conducted in Huh-6 cells after transfection of Linc00205 ov or NC. (**C**) Colony formation ability of Huh-6 cells were transfected with either Linc00205 ov or NC. Furthermore, we conducted quantitative analysis of colony formation (right panel). (**D**) Apoptosis assays of Huh-6 cells transfected with either Linc00205 ov or NC, as well as quantitative analysis of the apoptosis cells (right panel). (**E**, **F**) The migration and invasion ability of Huh-6 cells transfected with Linc00205 ov or NC were determined through the use of a transwell migration assay (**E**), and transwell invasion assay (**F**) (left panel). Quantitative analysis of the migratory cells or invasive cells in transwell assays was also performed (right panel). (**G**) Migration ability of Huh-6 cells transfected with Linc00205 ov or NC at indicated time points were evaluated by wound healing assay (upper panel). Quantitative analysis of the gap of wound healing assay (lower panel) was performed at 48 hours. *p < 0.05, **p < 0.01.

In order to further investigate the role of Linc00205 in HB, we downregulated Linc00205 by transfecting HepG2 cells using si-Linc00205, as HepG2 cells were found to have the highest Linc00205 expression across all HB cell lines. We also upregulated Linc00205 by transfecting Huh-6 cells with Linc00205 ov, as Huh-6 cells have the lowest Linc00205 expression across all HB cell lines. Next, we utilized qRT-PCR assays to determine transfection efficiency (Figures 2A, 3A, *p<0.05, **p<0.01). The CCK-8 assays exhibited that, in comparison with the negative controls, cell viability was found to decrease with Linc00205 knockdown (Figure 2B, *p<0.05), and increased with Linc00205 overexpression (Figure 3B, *p<0.05). Furthermore, we found that fewer colonies formed after knockdown of Linc00205 (Figure 2C, **p<0.01) and more colonies had Linc00205 overexpression (Figure 3C, **p<0.01) through colony formation assays. Flow cytometry results indicated that apoptotic rate increased when we downregulated Linc00205 (Figure 2D, **p<0.01), and decreased when Linc00205 was overexpressed (Figure 3D, **p<0.01). Both transwell migration assays and wound healing assays led to an impairment in cell migration in Linc00205 knockdown cells (Figure 2E, 2G, **p<0.01), as well as enhanced expression in Linc00205 overexpression cells (Figure 3E, 3G, **p<0.01). The transwell invasion assay verified that knockdown of Linc00205 impaired cell invasion (Figure 2F, **p<0.01), while overexpression of Linc00205 enhanced cell invasion (Figure 3F, **p<0.01). These results suggest that Linc00205 is a promoter of HB malignancy and suppressed Linc00205 is likely to suppress HB progression.

**Figure 4:**
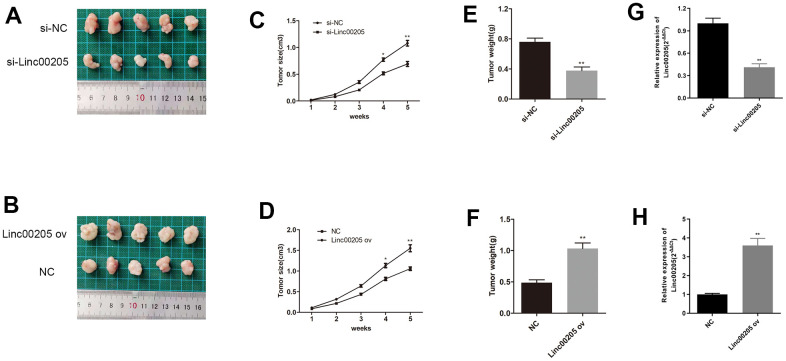
**Linc00205 expression affects HB cell-induced tumor formation.** (**A**, **B**) The representative appearances of the extracted xenograft tumors with implantation of si-Linc00205 transfected HepG2 cells (**A**) or Linc00205 ov transfected Huh-6 cells (**B**). (**C**, **D**) The size of tumors that were implanted with si-Linc00205 transfected HepG2 cells (**C**) or Linc00205 ov transfected Huh-6 cells (**D**), which were measured every seven days. (**E**, **F**) Quantitative analysis of weight of tumors with implantation of si-Linc00205 transfected HepG2 cells (**E**) or Linc00205 ov transfected Huh-6 cells (**F**). (**G**, **H**) qRT-PCR assay for Linc00205 expression in HepG2 cells that were transfected with si-Linc00205 or si-NC among xenograft tumors (**G**) or in Huh-6 cells transfected with Linc00205 ov or NC of xenograft tumors (**H**).*p < 0.05, **p < 0.01.

We also administered a subcutaneous injection of HB cells that were transfected with either si-Linc00205 or Linc00205 ov to mice in order to induce tumor formation. Tumors that were induced by si-Linc00205 transfected HepG2 cells were found to be significantly smaller in size, as well as lighter in weight, compared to tumors induced by si-NC-transfected HepG2 cells (Figure 4A, 4C, 4E). Furthermore, tumors that were induced by Linc00205 ov transfected Huh-6 cells were significantly larger in size and heavier in weight compared to tumors that were induced by NC transfected Huh-6 cells (Figure 4B, 4D, 4F). Lastly, we detected expression of Linc00205 in xenograft tumors through the use of qRT-PCR assays. We found that si-Linc00205 group had lower expression of Linc00205 compared to the si-NC group (Figure 4G), while Linc00205 ov group had higher expression of Linc00205 than the NC group (Figure 4H). Therefore, Linc00205 regulation may represent a novel therapy for HB patients.

### Linc00205 inhibited miR-154-3p as a sponge and inhibition of miR-154-3p restored HepG2 cells from Linc00205 knockdown

**Figure 5:**
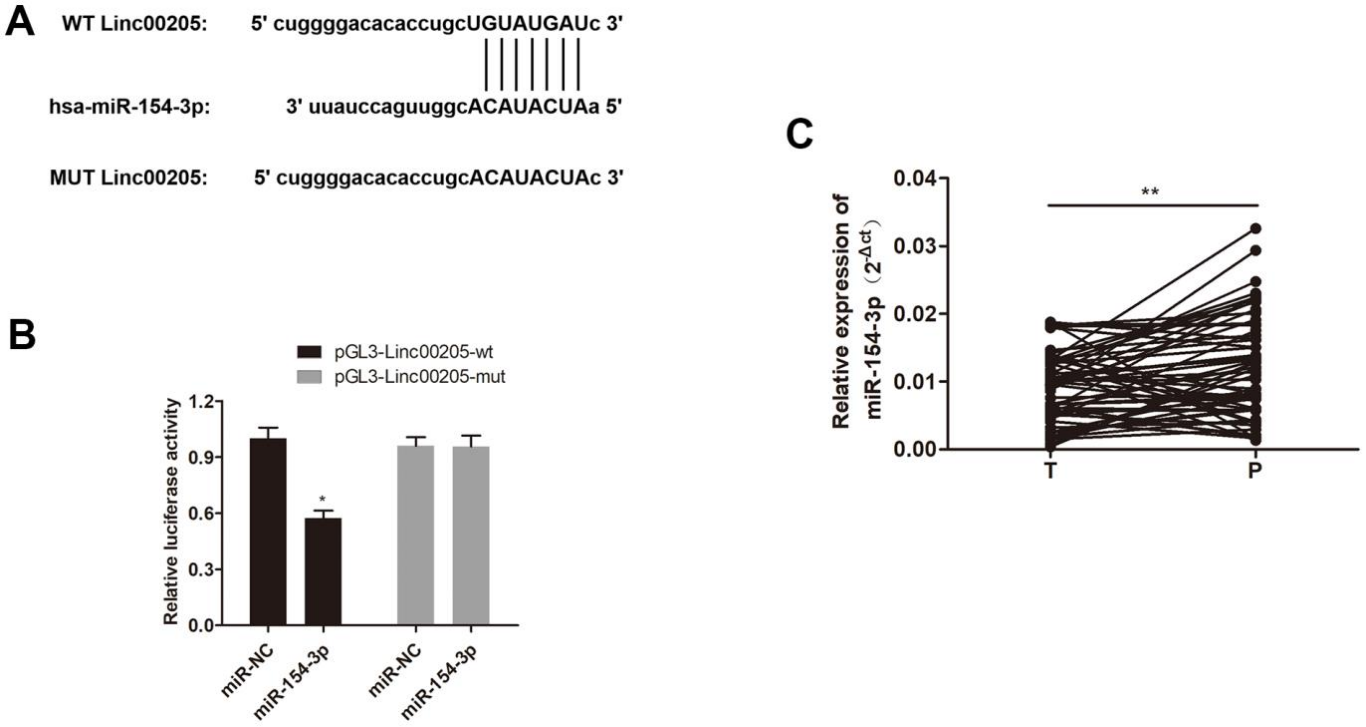
**miR-154-3p interacts with Linc00205 in HB cells.** (**A**) A schematic representation of the complementary sequence between Linc00205 and miR-154-3p. (**B**) Relative luciferase activity was examined in miR-154-3p and pGL3-Linc00205-wt or pGL3- Linc00205-mut co-transfected cells. (**C**) The qRT-PCR assays for miR-154-3p expression in HB tissues (n = 60) and para-cancerous tissues. *p < 0.05, **p < 0.01.

It has been previously reported thatLinc00205 interacts with miR-122-5p in HCC cell lines to promote growth [14]. Therefore, we explored possible Linc00205-targeted miRNAs, and discovered a complementary sequence between Linc00205 and miR-154-3p (Figure 5A), which suggests that miR-154-3p may possibly be a direct target of Linc00205. The dual-luciferase assay results validated this interaction, as luciferase activity was significantly reduced by pGL3-Linc00205-wt and miR-154-3p co-transfection, compared to the control group (Figure 5B, *p<0.05). Furthermore, qRT-PCR assay results demonstrated that expression of miR-154-3p in HB tissues was significantly downregulated, compared to the para-cancerous tissues (Figure 5C, **p<0.01). Which indicates that Linc00205 can directly sponge miR-154-3p, and that upregulation of Linc00205 can cause downregulation of miR-154-3p, which suggests a possibility that Linc00205 mediates HB progression by downregulating miR-154-3p.

**Figure 6:**
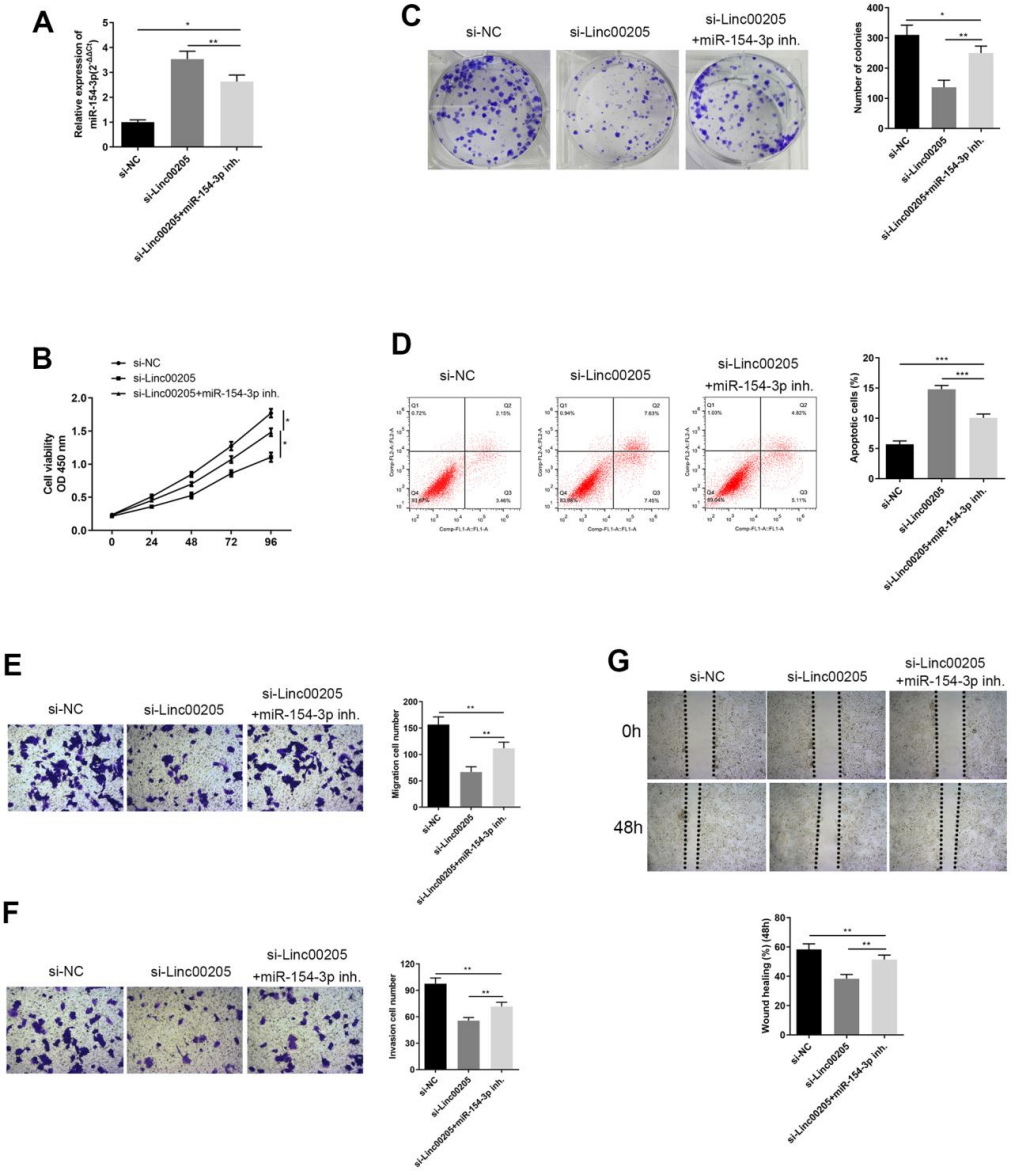
**Inhibition of miR-154-3p reversed changes in cell viability, colony formation ability, apoptosis, migration and invasion ability, caused by Linc00205 knockdown.** (**A**) qRT-PCR assay for miR-154-3p expression among HepG2 cells transfected with si-Linc00205 and with or without miR-154-3p inhibitor. (**B**) Cell viability assay was carried out in HepG2 cells that were transfected with si-Linc00205 and with or without the miR-154-3p inhibitor. (**C**) Colony formation ability of HepG2 cells were transfected with si-Linc00205 and with or without miR-154-3p inhibitor. A quantitative analysis of colonies was performed (right panel). (**D**) Apoptosis assays of HepG2 cells transfected with si-Linc00205 and with or without miR-154-3p inhibitor, and quantitative analysis of the apoptosis cells (right panel). (**E**, **F**) The migration and invasion ability of HepG2 cells transfected with si-Linc00205 and with or without miR-154-3p inhibitor were determined through the use of a transwell migration assay (**E**) and transwell invasion assay (**F**) (left panel). A quantitative analysis of migratory or invasive cells in transwell assays was also performed (right panel). (**G**) Migration ability of HepG2 cells transfected with either si-Linc00205 and with or without miR-154-3p inhibitor at indicated time points was evaluated by wound healing assay (upper panel). The quantitative analysis of the gap of wound healing assay (lower panel) was performed at 48 hours. *p < 0.05.

In order to confirm the regulatory role between Linc00205 and miR-154-3p, we inhibited miR-154-3p in Linc00205-knockdown HepG2 cells. miR-154-3p expression was significantly reduced in the si-Linc00205+miR-154-3p inh. Group, compared to the si-Linc00205 group (Figure 6A, p<0.01). Furthermore, cell viability significantly increased (Figure 6B, *p<0.05), and more colonies were formed (Figure 6C, p<0.01) compared to the si-Linc00205 group without miR-154-3p inhibitor. The apoptosis rate was also found to be significantly decreased with miR-154-3p inhibition, compared to the si-Linc00205 group (Figure 6D, p<0.01). Both the transwell migration assay and wound healing assay demonstrated cell migration (Figure 6E, 6G) and invasion ability (Figure 6F) of the Linc00205 knockdown cells, as miR-154-3p inhibition was significantly increased compared to the si-Linc00205 group. In conclusion, through the use of miR-154-3p inhibition, the malignancy that was suppressed by Linc00205 knockdown was restored. All these findings suggest that upregulated Linc00205 suppresses miR-154-3p regulatory role in HB cells, which causes cell carcinogenesis.

### ROCK1 is a target of miR-154-3p, which is dysregulated in HB tissues, and overexpression of ROCK1 restored HepG2 cells from miR-154-3p upregulation

**Figure 7:**
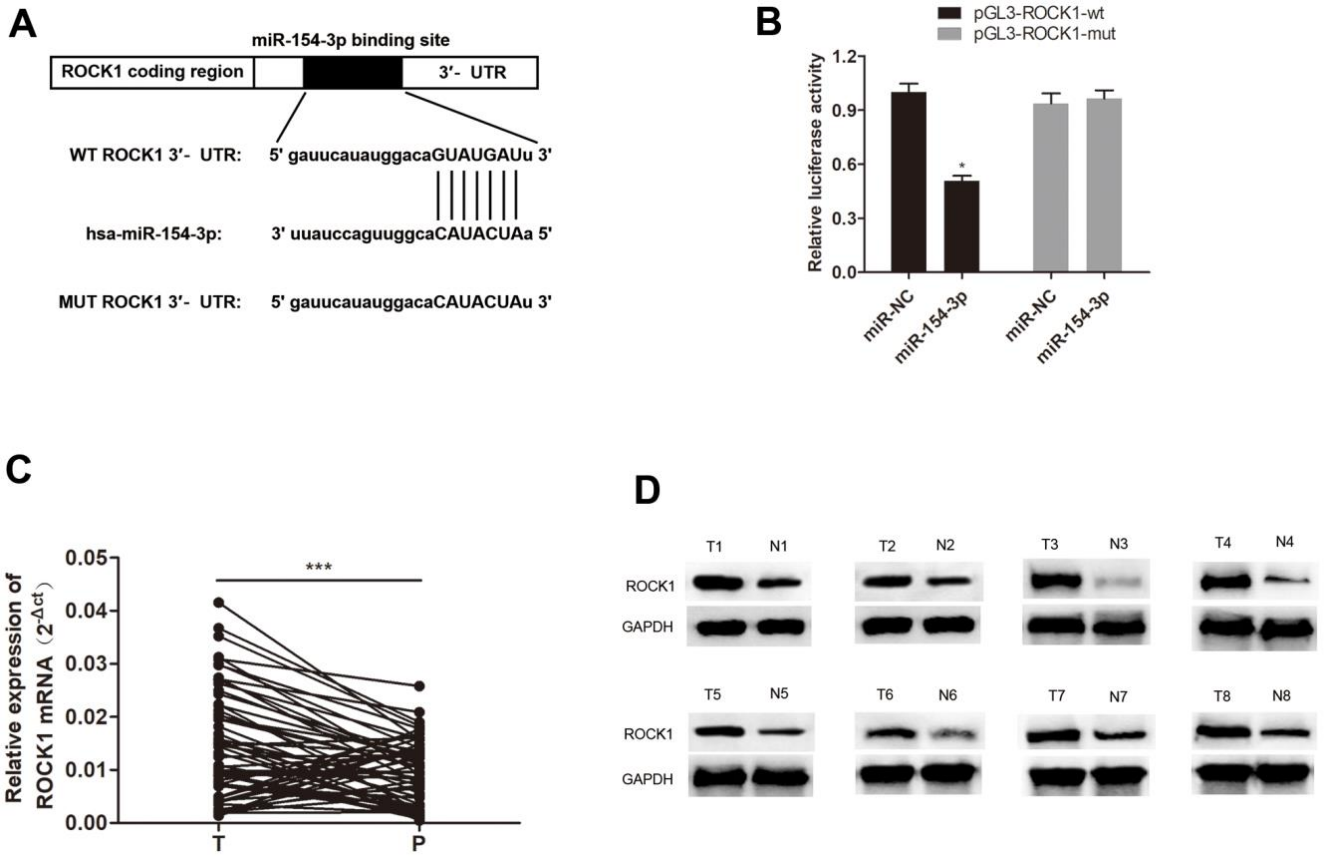
**miR-154-3p directly targets ROCK1.** (**A**) A schematic representation of putative miR-154-3p binding site in ROCK1. (**B**) Relative luciferase activity was examined in miR-154-3p and pGL3-ROCK1-wt or pGL3-ROCK1-mut co-transfected cells. (**C**, **D**) qRT-PCR and western blot assays for ROCK1 mRNA (**C**) (n = 60) and protein expression (**D**) (n = 8) in HB tissues and para-cancerous tissues.*p < 0.05, ***p < 0.001.

Furthermore, we investigated downstream target of miR-154-3p. Additionally, ROCK1 expression was found to negatively correlate with miR-154-3p [19], and a putative miR-154-3p binding site was discovered in the ROCK1 mRNA 3’UTR (Figure 7A), and significantly reduced luciferase activity was observed. Thus, we carried out a dual-luciferase assay and validated that miR-154-3p binds directly to ROCK1 by pGL3-ROCK1-wt and miR-154-3p co-transfection, compared to the control group (Figure 7B, *p<0.05). In addition, qRT-PCR and western blot assays for ROCK1 mRNA expression (Figure 7C, ***p<0.001) and ROCK1 protein expression (Figure 7D) in HB tissues were shown to be significantly higher than in para-cancerous tissues, which suggests ROCK1 may have a role in HB progression.

**Figure 8:**
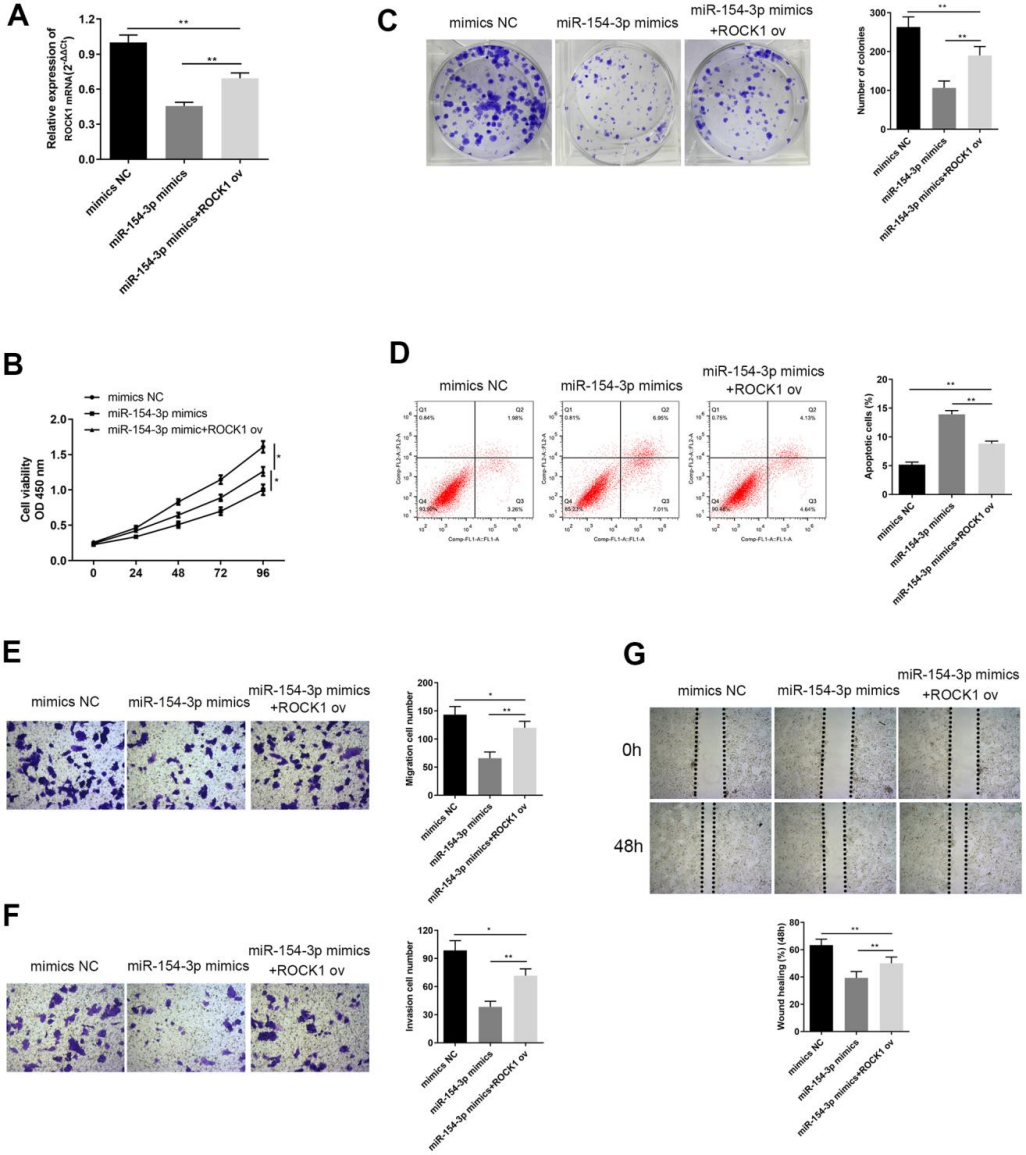
**Overexpression of ROCK1 altered changes in cell viability, apoptosis, colony formation ability, migration and invasion ability, caused by miR-154-3p mimics.** (**A**) qRT-PCR assay for ROCK1 mRNA expression in HepG2 cells, transfected with miR-154-3p mimics and with or without ROCK1 ov. (**B**) Cell viability assay was carried out in HepG2 cells transfected with miR-154-3p mimics and with or without ROCK1 ov. (**C**) Colony formation ability of HepG2 cells that were transfected with miR-154-3p mimics and with or without ROCK1 ov. Quantitative analysis was performed of the colonies were formed (right panel). (**D**) Apoptosis assays of HepG2 cells transfected with either miR-154-3p mimics and with or without ROCK1 ov, and quantitative analysis was performed of the apoptosis cells (right panel). (**E**, **F**) The migration and invasion ability of HepG2 cells transfected with miR-154-3p mimics and with or without ROCK1 ov were determined using the transwell migration assay (**E**) and transwell invasion assay (**F**) (left panel). Quantitative analysis of the migratory or invasive cells in transwell assays was also performed (right panel). (**G**) Migratory ability of HepG2 cells transfected with miR-154-3p mimics and with or without ROCK1 ov at indicated time points, as evaluated by wound healing assay (upper panel). Quantitative analysis of the gap of wound healing assay (lower panel) was performed at 48 hours. *p < 0.05.

In order to investigate the regulatory relationship between ROCK1 and miR-154-3p, we transfected HepG2 cells with a miR-154-3p mimic with or without ROCK1 ov. Co-transfection with ROCK1 ov remarkably increased ROCK1 levels, compared to the miR-154-3p mimics group (Figure 8A, p<0.01). Results of the CCK-8 assays showed that cell viability was enhanced by ROCK1 overexpression, compared to the miR-154-3p mimics group (Figure 8B, *p<0.05). Furthermore, more colonies were formed with ROCK1 overexpression in colony formation assays, compared to the miR-154-3p mimics group (Figure 8C, p<0.01). Flow cytometry results suggested that there were fewer apoptotic cells with ROCK1 overexpression, compared to the miR-154-3p mimics group (Figure 8D, p<0.01). The transwell migration assay (Figure 8E, p<0.01) and wound healing assay (Figure 8G, p<0.01) both demonstrated that the cell migration ability was enhanced with ROCK1 overexpression, compared to the miR-154-3p mimics transfected cells, and the capacity was restored to level of the mimics NC group. Invasion ability was also enhanced with ROCK1 overexpression, compared to the miR-154-3p group (Figure 8F, p<0.01). These results suggest that miR-154-3p can downregulate ROCK1 function, and have an essential role in inhibiting HB development.

### Linc00205 affects HB cell malignancy by modifying p38 MAPK signaling pathway-related protein through regulating ROCK1 and may further affect related proteins of the EMT pathway, apoptosis pathways, and cell cycle

**Figure 9:**
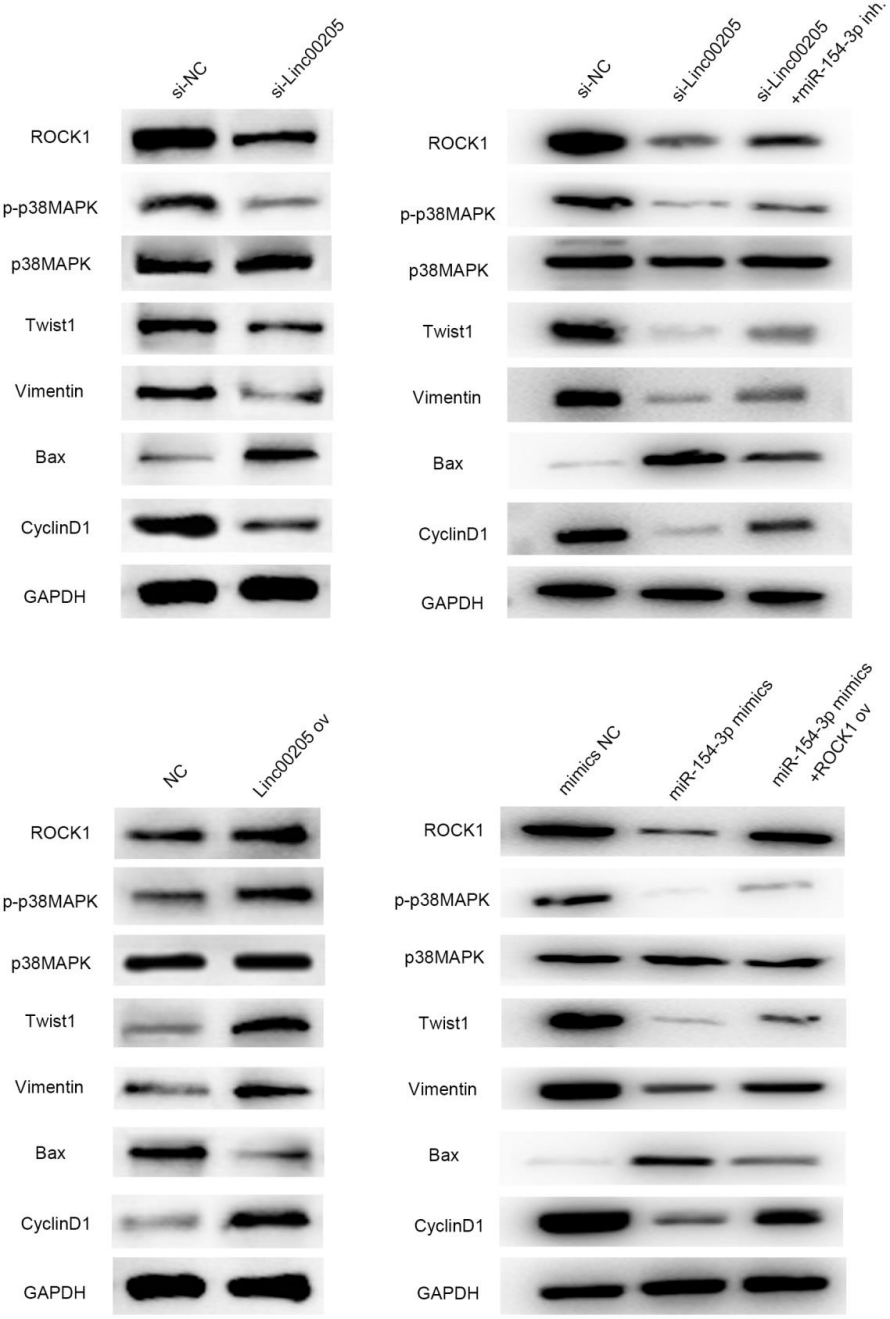
**Linc00205 affects the p38 MAPK signaling pathway by interacting with ROCK1, and further affects EMT pathway-related protein (vimentin), apoptosis pathway protein (Bax) and proliferation pathway protein (cyclinD1) and the effects are regulated by miR-154-3p and ROCK1.** Western blot assays for ROCK1, p-p38MAPK, p38MAPK, Twist, Vimentin, Bax and CyclinD1 protein expression in HepG2 cells transfected in different groups.

Next, we needed to investigate the downstream pathways that were affected by dysregulation of Linc00205. Thus, western blot assays were performed to evaluate the protein expression of several proteins involved in cellular functions. The results demonstrated that the phosphorylation of p38 was impaired when Linc00205 was knocked down, and upregulated when Linc00205 was overexpressed (Figure 9). In addition, p38 is an essential member of the p38 MAPK signaling pathway [20]. Twist1 expression and EMT pathway-related protein Vimentin [21] were positively correlated with Linc00205 expression. The expression of cell cycle-related proteins, including cyclinD1 [22], was positively correlated with Linc00205, while expression of Bax, the apoptosis pathway-related protein [23], was negatively correlated with Linc00205 expression. Besides, the western blot results revealed that miR-154-3p inh. could rescue the effect induced by si-Linc00205 on these proteins, and ROCK1 ov could impaired the effect of miR-154-3p mimics. All results indicated that regulating Linc00205 can interact with miR-154-3p and ROCK1 to help produce a massive impact on tumor progression and metastasis through the MAPK signaling pathway, EMT pathway, cell cycle-related pathway and apoptosis-related pathway.

## DISCUSSION

HB is a common malignant pediatric liver tumor, and the prognosis of HB varies according to different categories. The practical methods for treating HB are limited, and mainly include surgery and chemotherapy. However, HB has significant tumor heterogeneity, as it is formed by tumors that are derived from different immature liver precursors, including biliary, hepatocytes, and other mesenchymal or epithelial cells [24]. Therefore, it is essential to explore deeper into the underlying molecular mechanism of HB. Herein, we, for the first time, identified Linc00205 as an oncogene of HB that promotes HB malignancy by regulating metabolic pathways. The Linc00205/miR-154-3p/ROCK1 axis represents a novel target for HB-targeted therapy.

LncRNAs have attracted a lot of attention in recent years for their potential in lncRNA-based cancer therapy. Cancer treatments that target lncRNAs are thought to be novel and promising therapy with high specificity, collective therapeutic efficacy, as well as fewer side effects [25]. Several studies have suggested that dysregulated expression of lncRNAs can be used as novel molecular biomarkers in cancer diagnosis and prognosis prediction [26, 27]. Herein, we identified that Linc00205 is an oncogene, is significantly highly expressed in HB tissues and cells, and high Linc00205 levels are negatively correlated with a five-year survival rate. Furthermore, knockdown of Linc00205 expression contributes to inhibition of HB cell proliferation, migration and invasion *in vitro*, as well as reducing tumor volumes *in vivo*.

It also has been widely accepted that lncRNAs can act as a miRNA sponge, and form a lncRNA-miRNA-mRNA network with miRNAs in order to regulate target protein expression [28]. For example, the LncRNA SPRY4-IT1 can sponge miR-101-3p, and therefore up-regulate EZH2 expression, which promotes proliferation and metastasis of bladder cancer cells [29]. This study shows that Linc00205 sponges miR-154-3p in order to promote proliferation and metastasis of HB cells by upregulating ROCK1.

The prominent role of the ROCK1 pathway is to regulate cell movement. ROCK1 activation increases phosphorylation of the myosin light chain (MLC) of myosin II, which ultimately increases cell contractility. ROCK1 also participates in phosphorylation of LIMK1/2, which can help increase LIMK catalytic activity and facilitate re-arrangement of the actin cytoskeleton [30]. ROCK1 also participates in regulating cell cycle-related proteins that increase expression of cyclins A/D1/D3 [31], where regulation of cyclin D is thought to be indirectly regulated by activation of Ras/MAPK pathway [32]. The interaction between ROCK1 and MAPK signaling has also been reported by several studies. For example, regulation of RhoC/ROCK1/MAPK axis likely alters breast cancer cell progression [33]. The LncRNA AFAP1-AS1 promotes tumorigenesis and epithelial-mesenchymal transition of osteosarcoma through the RhoC/ROCK1/p38MAPK/Twist1 signaling pathway [34]. The MAPK pathway is an important nodal pathway point that the signaling node not only functions as a tumor suppressor, but also as a pro-oncogenic signal. The p38γ MAPK pathway has been suggested to mediate EMT of breast cancer through the p38γ MAPK/GATA3/miR-200b/Suz12 [35]. Translocation of Bax to the mitochondria also requires activation of p38 MAPK, which causes apoptosis [36]. Therefore, cell cycle, apoptosis and EMT pathways are likely all downstream regulatory targets of the MAPK pathway. Herein, ROCK1 was considered to be a target of miR-154-3p, and showed that ROCK1 expression is positively correlated to Linc00205 levels in HB. Our results also demonstrated that Linc00205 facilitates HB progression by competitively binding miR-154-3p in order to activate ROCK1-mediated MAPK pathway, in order to promote EMT.

## CONCLUSIONS

In conclusion, we identified Linc00205 as an oncogene that had a crucial role in the proliferation and metastasis in HB. Additionally, our study identified a Linc00205/miR-154-3p/ROCK1 axis for the first time. We also revealed that Linc00205 positively regulated posttranscriptional expression of ROCK1 by sponging miR-154-3p in HB. Furthermore, knockdown of Linc00205 attenuated oncogenic function and decreased ROCK1 expression, which led to an inhibition of the MAPK pathway, thereby providing a novel therapeutic target in HB. Clinically, the detection and regulation of the Linc00205/miR-154-3p/ROCK1/MAPK axis may play an important role in diagnosis and treatment of HB.

## MATERIALS AND METHODS

### Patient samples

In total, 60 pairs of HB tissues and para-cancerous tissues were collected from HB patients at the Children’s Hospital of Nanjing Medical University. The inclusion criteria comprised of (1) patients must be diagnosed with HB with relevant clinical diagnosis, and (2) patients must not have received any anti-tumor treatment prior to surgery. The exclusion criteria indicate that patients with organic diseases and other tumors were excluded. After extraction, the tissues were frozen and stored in a -80° C freezer till the experiments were carried out. All related patients were informed about the research, and signed informed consent letters. The experiments were conducted in agreement with the ethical standards at the ethics committee of Children’s Hospital of Nanjing Medical University, as well as the 1964 Helsinki Declaration.

### Cell lines and cell culture

The HB cell lines, including HepG2, SMMC-7721, Huh-6, and the normal human hepatic cell line L02 were cultured in 10% FBS DMEM medium in an environment of 5% CO_2_ and 37° C.

### Cell transfection

Small interfering RNAs against Linc00205 (si-Linc00205), as well as small interfering RNA negative control (si-NC), were cloned into PGK plasmids and used to knockdown expression of Linc00205 in the HepG2 cell line. The pcDNA 3.1 plasmids with full length Linc00205 (Linc00205 ov) and related negative controls (NC) were utilized to upregulate Linc00205 in the Huh-6 cell line. The miR-154-3p inhibitor and mimics were also utilized to regulate expression of miR-154-3p. The pcDNA3.1 plasmids with full length of ROCK1 (ROCK1 ov) and related negative control (NC) were utilized to upregulate ROCK1 in HepG2 cell line. All factors were transfected into cells using Lipofectamine™ 2000, and adhered to the provided protocol.

### Quantitative real-time PCR (qRT-PCR)

Total RNA in HB tissues and cells was extracted using the TRIzol reagent. RNA was reverse-transcribed into cDNA. Linc00205 and ROCK1 expression were detected using ABI 7900HT RealTime PCR System through the use of SYBR Green assays. GAPDH was used as internal control. The expression of miR-154-3p was quantified via TaqMan MicroRNA Assays and U6 was treated as internal control. The 2^−ΔCt^ and 2^−ΔΔCt^ method helped evaluate RNA expression.

### Western blot

Whole-cell lysate was prepared using the RIPA protein solubilization buffer. Protein levels were quantified utilizing the BCA kits and blocked using 5% skim milk at room temperature for one hour. After washing with PBS three times, the proteins were incubated with primary antibodies (ROCK1, p-p38MAPK, p38MAPK, Twist, Vimentin, Bax, cyclinD1, and GAPDH) overnight at 4° C. The membrane was then washed with PBS three times for 5 minutes each time, which was followed by incubation with secondary antibody (1:2000) and incubation at 25° C for 2 hours. Finally, the band was visualized using ECL luminescence solution in a dark room. Images were developed and analyzed using the ChemiDocXRS+ system.

### Cell proliferation detection

Cell viability assay was conducted using the Cell Counting Kit-8 (CCK-8), which followed manufacturer’s instructions. Results were determined by measuring light absorbance at 450 nm on a microplate reader. Colony formation assay was carried out by culturing 500 HB transfected cells in 6-well plates, respectively, for two weeks. The colonies were then fixed using 4% paraformaldehyde for 20 minutes, and then stained with 0.1% crystal violet for 30 minutes. Finally, the colonies were quantified using the Image J software.

### Cell migration and invasion

Transwell assay was utilized to determine cell migration and invasion ability of HB cell lines. Cells were placed into the top chamber of a transwell with serum-free media. At the same time, the lower chamber was filled with complete medium. The filters that were used for invasion assays were then coated with 30 μL pre-diluted Matrigel. After 24 hours of incubation at 37° C, the migrated or invaded cells were stained using 0.1% crystal violet, and then observed under a microscope. Cells that migrated into the lower chamber were visualized and counted.

Wound healing assay was utilized to determine the cell migration ability of HB cell lines. A 200 μL pipette tip helped create a scraped area in a transfected cells culture dish. Next, cells were washed twice and cultured in medium with 0.1% FBS in order to eliminate cell proliferation effect. The healing distance was photographed at 0 and 48 hour time points.

### Cell apoptosis evaluation

Cell apoptosis was detected via Annexin V-FITC/PI double staining, and analyzed using flow cytometry. Cells were harvested and washed with PBS, and then resuspended in 500 μL binding buffer, which contained 5 μL of Annexin V-FITC and 5 μL of propidium iodide. After 10 minutes of incubation in the dark at room temperature, samples were then applied for flow cytometry (FCM) analysis.

### Dual-luciferase reporter assay

HepG2 cells were seeded onto 48-well plates until they reached 70% confluence. Each well was transfected using 100 ng luciferase vector, 400 ng miR-154-3p mimics or mimics NC, and pGL3-Linc00205-wt or pGL3-Linc00205-mut plasmid, pGL3-ROCK1-wt or pGL3-ROCK1-mut plasmid. After the cells were cultured for 24 to 48 hours, cells were washed twice with PBS. With the dual-luciferase reporter assay kit’s material and protocol, cells were lysed with 150 μL lysate. In addition, 30 μL cell lysate was applied to the special black enzyme-labeled hole with addition of LAR II detection buffer for luminescence detection. After adding detection termination solution to each well, luminescence detection was carried out again. Finally, the values were derived and calculated.

### Xenograft subcutaneous tumor formation assay

Five-week old male BALB/C mice were stored at 22-24° C routinely and administered dorsal subcutaneous injection of HepG2 cells transfected with si-Linc00205 or Huh-6 cells that were transfected with Linc00205 ov, suspended in 100 μL of Matrigel. Tumor volume was examined every seven days. The calculation formula was as follows: volume =0.5× length × width × height. Then, five weeks after the injection, animals were euthanized and their tumor weights were measured. Animal experiments were granted approval by the Animal Ethics Committee of Children’s Hospital of Nangjing Medical University.

### Statistical analysis

Statistical analysis was carried out using the SPSS software (Version 20.0) and graphpad7. Data were presented as mean ± standard deviation (x±s) of at least three biological replicates. A *t*-test was utilized for the intergroup comparisons, and a one-way analysis of variance was used to compare between groups. Values of p<0.05 indicated statistical significance.
